# Unexpected Intraspecific Variability of Perennial Ryegrass (*Lolium perenne* L.) in Response to Constant Temperature During Germination and Initial Heterotrophic Growth

**DOI:** 10.3389/fpls.2022.856099

**Published:** 2022-04-08

**Authors:** Lina Q. Ahmed, Abraham J. Escobar-Gutiérrez

**Affiliations:** INRAE, URP3F, Lusignan, France

**Keywords:** breeding, dormancy (seed), germinability, heterotrophic growth, relative growth rate (RGR)

## Abstract

*Lolium perenne* (L.) is one of the most important species in temperate grasslands. Temperature is a major factor controlling plant development. Breeding *L. perenne* cultivars adapted to new ranges of temperature could be necessary under most climate change scenarios. However, before any breeding effort in such direction, knowing the intraspecific variability of responses to temperature is essential. Our objective was to analyze a sample of accessions of *L. perenne* for their response to constant temperature during germination and initial heterotrophic growth. Eight accessions were taken out from a genebank containing 160 accessions. Six accessions were wild populations collected in different places in France and the other two were populations from a selection program. For germination, four replicates of 100 seeds per accession were tested in Petri dishes in the dark at eight constant temperatures, from 5 to 40°C with increases of 5°C. Germination countings were carried out at variable time intervals and durations that depended on treatments. For initial heterotrophic growth analysis, seeds were germinated at 25°C. Sets of 30 seedlings per accession were placed in polypropylene boxes on blotter blue paper and transferred to each one of the eight treatments in the dark. They were pictured at variable time intervals for root and shoot growth measurement by image analysis. Neither seed germination nor heterotrophic growth was observed at 40°C, for any accession. Temperature and time course response surfaces were markedly different between accessions. Equally, maximum germinability and the shape of the response curves to temperature were significantly different between accessions. This means that limited similarities between responses were observed. Furthermore, germination rates followed the non-linear beta function with significant differences between some accessions. These also showed significant differences in their root and shoot growth rate in response to temperature. In general, the relative growth rates of roots and shoots were slow at 5°C, peaked between 25 and 30°C, and showed a sharp reduction afterward. These results reveal, for the first time, high genetic variability within *L. perenne* germplasm for the response to temperature in the initial life phases. This discovered variability should serve breeders to create perennial ryegrass varieties for the future.

## Introduction

Perennial ryegrass (*Lolium perenne* L.) is the most important forage grass species sown in temperate agriculture systems where it is exploited *via* grazing and mowing. Its economic value is based on the high quality and quantity of the biomass produced (Smith et al., 2001; Baert and Van Waes, 2014). Since the 1960s, agronomic research centers and private firms have developed breeding programs to release improved cultivars of forage species that ensure high production of forage of good quality (Sampoux et al., 2011). Knowing the intraspecific phenotypic and genotypic variability is essential for any selection and breeding program. Despite the high genetic diversity of this species in the wild, the genetic basis of modern cultivars is extremely narrow (Blanco-Pastor et al., 2019; Faville et al., 2020).

Climate change in Europe is expected to affect the local annual courses of precipitation and temperatures. It is becoming a major threat to natural and cultivated grasslands. Indeed, recent studies suggest that environmental local changes will be faster than the adaptation of natural populations of grasses (Keep et al., 2020). Furthermore, the performances of the current panel of commercial cultivars could be also affected. Thus, one of the current challenges of forage breeders is to create new cultivars adapted to the future climate conditions. To this end, exploiting the genetic diversity found in wild populations could be of paramount importance (Blanco-Pastor et al., 2019; Keep et al., 2020).

Temperature is one of the major factors controlling plant development such as seed germination and plant growth. Thus, breeding perennial ryegrass cultivars adapted to new ranges of temperature could be necessary under most climate change scenarios. However, before any breeding effort in such a direction, an account of the intraspecific variability of responses to temperature is essential (Ghaleb et al., 2021, 2022). Seed germination (Ahmed et al., 2019; Ghaleb et al., 2021) and early heterotrophic growth (Escobar-Gutiérrez et al., 1998a,b; Ahmed, 2015) in response to temperature can be used as an early marker for selecting promising material.

Interspecific comparisons in the responses to temperature during germination have been extensively reported. Actually, numerous studies have explored the relation between temperature and germination in forage grasses (Baskin and Baskin, 2014). Some of them report comparisons between *L. perenne* and other species [e.g., Larsen and Bibby (2005); Zhang et al. (2013), and Lin et al. (2018)]. However, surprisingly, nothing is known on the natural intraspecific diversity of responses, if any, to temperature during germination of *L. perenne* and early growth. Indeed, to the best of our knowledge, only two published studies on this species have focused on comparing commercial cultivars under alternating and constant temperatures (Larsen and Bibby, 2005; Shen et al., 2008).

Our overall aim is to explore the genetic diversity of *L. perenne* to find sources for breeding. The objective of this study was to analyze a sample of accessions of *L. perenne* for their response to constant temperature during germination and initial heterotrophic growth.

## Materials and Methods

### Plant Material

Eight accessions of *L. perenne* L. were used in this study. Six of them are wild populations collected in different sites in France (Charmet et al., 1990). Two are populations from a breeding program at INRAE, Lusignan, France (Table 1). The wild populations are a sample from the 160 accessions of *L. perenne* conserved in Centre de Ressources Biologiques des Espèces Fourragères in Lusignan, France (46°24‘15″N, 0°04‘45″E)^1^. The six French wild populations were chosen considering their provenance (North, South, mountains, and plains) and seed availability. They were stored in opaque envelopes in the dark at 5°C and 30% relative humidity (RH) until they were used. Initial seed dry weight (SDW) was determined in four replicates of hundred seeds for each accession.

**TABLE 1:** List of six wild populations of *Lolium perenne* L. collected in different sites in France and two populations from a selection program (P19 and H1) obtained by divergent selection at INRAE-URP3F, Lusignan, France.

Accession	Altitude	Collection site	Latitude and longitude	Mean temperature coldest quarter (°C)	Mean temperature warmest quarter (°C)	Precipitation coldest quarter (mm)	Precipitation warmest quarter (mm)
ACVF10214	275	Nailloux	43°21’24.10″N, 1°37’24.10″E	5.2	20.0	185	170
ACVF10491	541	Bésignan	44°19’14.63″N, 5°19’30.93″E	2.6	18.3	202	171
ACVF20010	343	Bosdarros	43°12’35.71″N, 0°21’43.34″W	5.1	18.4	241	188
ACVF50013	544	Saulieu	47°16’46.18″N, 4°13’43.25″E	1.4	16.7	229	226
ACVF50039	322	Lure	47°41’11.10″N, 6°29’39.39″E	1.5	17.5	259	250
ACVF60016	99	Reims	49°15’29.98″N, 4°01’54.11″E	2.7	17.6	175	149
P19	153	Le Chêne	46°24’40″N, 0°12’21.93″E	4.4	18.3	18.3	162
H1	Idem	Idem	Idem	Idem	Idem	Idem	Idem

*Information on the collection sites is included.*

### Seed Germination Experiments

The protocol was first described in Ahmed (2015). Two experiments were conducted as follows. In the main experiment, four repetitions of one-hundred seeds per accession were tested for germination in the dark at eight treatments of constant temperature from 5 to 40°C, with 5°C increments (Table 2). The one-hundred seeds of each repetition were imbibed on top of two sheets of sterilized Whatman paper (ref. 3645 Whatman, France) in 90 mm diameter sterilized polyethylene Petri dishes containing 5 ml of deionized and autoclave-sterilized water. At each temperature, the design was a randomized complete block with four replicates. The dishes of each block were placed in a vented plastic box. Seeds were cold stratified in the dark for 1 week at 5°C and 30% RH, to break any residual dormancy. After cold stratification, seeds were germinated in the dark at constant temperatures in growth chambers. To reduce the risk of having effects induced by the chambers (“chamber effect”), temperature and RH in the chambers were recorded every minute during the experiment. Furthermore, the temperature within the useful volume of the chambers was checked by six thermocouples placed within the vented plastic boxes at different positions around the Petri dishes and logged every 20 s.

**TABLE 2:** Temperatures, relative humidity, and vapor pressure deficit (VPD) were measured for germination.

Temperature treatment (°C)	Temperature actual (°C)	Relative humidity %	VPD (kPa)	Sampling frequency (h)	Duration of observations (h)
5	5.0	74	0.23	48	2,352
10	9.6	84	0.20	16	1,584
15	14.3	76	0.41	12	900
20	19.2	74	0.61	8	952
25	25.0	80	0.63	8	896
30	30.0	50	2.12	8	640
35	34.2	65	1.97	12	732
40	40.0	55–100	3.32–0.00	168	1,680

A seed was considered as germinated when the radicle or the coleoptile had protruded out of the seed and was at least 2 mm long (Bewley and Black, 1994). At each counting, germinated seeds were removed from the Petri dishes, and autoclave-sterilized water was added to ensure non-limiting moisture. Because germination speed varies with temperature in a non-linear way (see germination modeling below), germination counts were carried out at variable time intervals (8–168 h) and duration of the observations (640–2,352 h) that depended on treatments (Table 2).

In the experiment described above, no germinated seeds were observed at 40°C for any accession, even after several weeks. Furthermore, under high temperature and humidity, saprophytic fungi developed that made it impossible to follow the seeds beyond 12 weeks. Consequently, a subsidiary experiment was conducted at 40°C under aseptic conditions, to exclude any artifact in the main experiment at this temperature. To this end, seeds were counted under a laminar flow hood and sterilized by soaking them in a 2.5% sodium hypochlorite solution for 10 min. Seeds were then rinsed three times with running autoclave-sterilized water. Polypropylene boxes (55 mm × 120 mm × 180 mm, GEVES trademark, Loire Plastic, France, called GEVES-type box), sterilized with ethanol 99%, were used for this experiment. Sheets of Whatman paper were wrapped in aluminum foil and sterilized in an autoclave at 120°C for 20 min. Furthermore, paper sheets were dried at 80°C for 48 h. Seeds were laid over four sheets of Whatman papers in a GEVES-type box with 15 ml of deionized and autoclave-sterilized water. The boxes were wrapped in aluminum foil and transferred to 5°C and 30% RH for 7°days for cold-stratification, as above. After stratification, seeds were transferred into a walking-in growth chamber at 40°C and vapor-pressure deficit under 1°kPa. Temperature and RH in the chambers were recorded every minute during the experiment.

### Initial Heterotrophic Growth Experiments

After cold stratification, seeds were installed for germination at 25°C as described above. Seedlings with radicle or coleoptile longer than 1 mm were chosen and transferred to be grown at the eight temperature treatments. For each accession, three groups of ten seedlings each were placed in GEVES-type boxes on blotter bleu paper (Anchor Paper, St. Paul, Min) and watered with 20 ml deionized and autoclave-sterilized water as needed. Each seedling was numbered and its growth followed. For the experiment at 40°C, seeds and materials were sterilized as described above. The boxes were placed on the long side with 90° and 60° angles at 5–35 or 40°C, respectively, in growth chambers. Boxes inclinations were meant to keep seedlings growing upright, without bending away from the blotter paper.

For measurement of axes elongation (radicle and shoot), pictures were taken routinely using a Nikon D70 digital camera with an objective Micro Nikon 60 mm-f/2.8 in automatic mode and sensibility set to 200 ISO. The camera produced images with a resolution of 3,008 pixels × 2,000 pixels. Pictures of the seedlings were taken at variable time intervals and duration that depended on temperature treatment, similar to germination follow-ups (Table 2). For each accession per temperature, 90 photos were taken, 30 photos per replicate.

The first picture of each replicate by accession at 25°C was used to determine seed length (SL) and width (SW).

Images were analyzed using ImageJ software (version 1.47).^2^ Length measurements were converted into millimeters using an internal standard. Images were analyzed using a combination of automated and manual steps.

### Calculations

#### Germination Modeling

For each accession, data were normalized for its maximum germination observed in any Petri dish, regardless of temperature treatment. This maximum value represented the potential of germinability of the accession. We considered that normalized data were the best base for response comparative purposes.

As in Ghaleb et al. (2021), for each replicate, the cumulated number of germinated seed was fitted, using the least-squares method (Steel and Torrie, 1980), to a non-rectangular hyperbole (Equation 1) of the form:


(1) $$\begin{array}{c} y=\left(\frac{1}{2\theta }\right)(\alpha \cdot \left(t-tc\right)+ymax \end{array}$$

where *y* is the cumulated germination (% of seeds); θ is a parameter that determines the shape of the curve (unitless); α is the maximum germination rate (% of seeds per hour or % of individuals per hour); *t* is time (hour); *tc* is the lag to start germination (hour); and *y*max is the maximum germination (% of seeds) or germinability. Excellent fits were obtained with this mathematical function. An advantage of such a model is the possibility of attributing eco-physiological meaning to the parameters (Escobar-Gutiérrez et al., 2009).

From Equation 1, it follows that the time to get 50% of the potential cumulated germination, *t*_50%_, equals:


(2) $$t_{50\%}=\left[\frac{tc+ymax-\frac{\left(ymax.\theta \right)}{2}}{\alpha }\right]\;$$

Finally, time the interval between *tc* and *t*_50%_ is called **τ**.

For each accession, the normalized maximum germination percentages observed in the four replicates were pooled together and plotted against temperature. Third-degree polynomials were fitted to each data set and optimal temperature for germination was calculated as:


(3) $$x=\frac{-b\text{±}\sqrt{b^{2}-4ac}}{2a}\;$$

where *x* is the optimal temperature, *a*, *b*, and *c* are the parameters of the polynomial.

The α_50%_ was fitted a five parameters beta model, either Equations 4 or 5:


(4) $$y=y\max\cdot {\left({\left(\frac{\left(T-T\text{min}\right)}{To-T\text{min}}\cdot \frac{\left(T\text{max}-T\right)}{T\text{max}-To}\right)}^{\frac{T\text{max}-T}{To-T\text{min}}}\right)}^{\beta }$$

(5) $$y=y\text{max}\cdot {\left({\left(\frac{\left(T\text{max}-T\right)}{\left(T\text{max}-To\right)}\cdot \frac{\left(T-T\text{min}\right)}{\left(To-T\text{min}\right)}\right)}^{\frac{To-T\text{min}}{T\text{max}-To}}\right)}^{\delta }$$

with the three cardinal temperatures, *T*_*min*_, *T*_*o*_, and *T*_*max*_; the rate maximum rate *y*_*max*_ at *T*_*o*_ and a shape parameter either *ß* or δ (Yin et al., 1995).

#### Initial Heterotrophic Growth Modeling

The kinetics of growth of radicle and shoot of each one of the 30 seedlings per accession were fitted, using the least-squares method (Steel and Torrie, 1980), to the Schnute’s non-linear model (Equation 6) (Schnute, 1981):


(6) $$y={\left(c^{b}+\left(d^{b}-c^{b}\right)\cdot \frac{1-exp^{\left(-\alpha \left(t-v\right)\right)}}{1-exp^{\left(-\alpha \left(w-v\right)\right)}}\right)}^{\frac{1}{b}}\;$$

where the dependent variable is *y* (mm); unitless parameters *a* and *b* implicitly define the shape of the curve; parameters *c* (mm) and *d* (mm) are the lower and upper values of *y* at *v* and *w*, respectively; *v* (hour) and *w* (hour) are, respectively, the initial and last time of the fitted growth period (Ahmed, 2015).

The first and second derivatives of Schnute’s adjustments were used to estimate the maximum growth rates (MGR) in mm.h^–1^ of the two axes for each seedling. They allow also estimating the time, *t*, at which MGR occurs.

To estimate the relative growth rate (RGR) at the time, *t*, when MGR occurs, we used Equation 7 such that:


(7) $$\text{RGR}\left(t\right)=\mathrm{MGR}\left(t\right).\frac{1}{y\left(t\right)}\;$$

where RGR(*t*) is the relative growth rate at time *t*, MGR(*t*) is the maximum growth rate (mm.h^–1^) at time *t*, and *y*(*t*) is the length of the axes (mm^–1^) at time *t*. For each accession, the relative elongation rate of axes (mm.h^–1^) in response to temperature were pooled together and plotted against temperature. The five parameters beta model (Equations 4, 5) was fitted to each data set. The minimum temperature for growth, the low boundary, was fixed at 0°C, under the assumption that no vascular plant can grow below 0°C (Körner, 2016).

### Statistical Analyses

Statistical analyses were performed to estimate the contribution of accession’s effects on the variability of results and in response to temperature. Sequential pair-wise comparisons were performed between the best fits of accession *i* and the raw data of accessions one to eight. Concerning germination, these tests were performed for maximum germination percentage (polynomials) and for the germination rate when half of the seeds have germinated, α_50%_ (beta model), in response to temperature. For each couple of model-data compared, a two-sided Shapiro–Wilk test (*Sh-W. test* procedure) was first applied to verify the normal distribution of residuals when switching datasets. A Student’s test (*t-test*) was then applied to determine any bias of the mean between the two models (mean difference assumed equal to 0). Finally, when the two previous tests were successful, a lack-of-fit test (*pf* procedure) was performed to test for significant differences between the model fits (Steel and Torrie, 1980). The probability of a calculated *F value* is greater than that of a tabular *F* (*Pr > F*) was calculated and comparative matrices were constructed (*P < 0.05*).

Concerning initial heterotrophic growth, the normal distribution of residuals of the sequential pair-wise comparisons was assessed with the Kolmogorov–Smirnov test.

All these statistical analyses were performed with R language (R version 3.5.1 (2018-07-02),^3^ packages: base, stats, and nlstools. R Core Team, 2021).

## Results

### Temperature and Time Course Response-Surfaces

Data on maximum germination percentage (germinability) as a function of temperature were analyzed. For each accession, data were normalized for the maximum germination observed in any Petri dish, regardless of temperature treatment. These normalized data were then extensively used and are presented hereafter.

**FIGURE 1:**
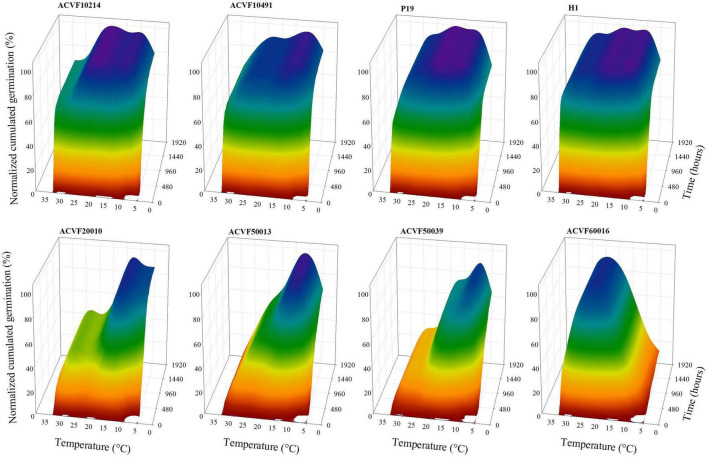
Temperature and time course response surfaces of the normalized cumulative germination percentage of eight accessions of *Lolium perenne*.

**FIGURE 2:**
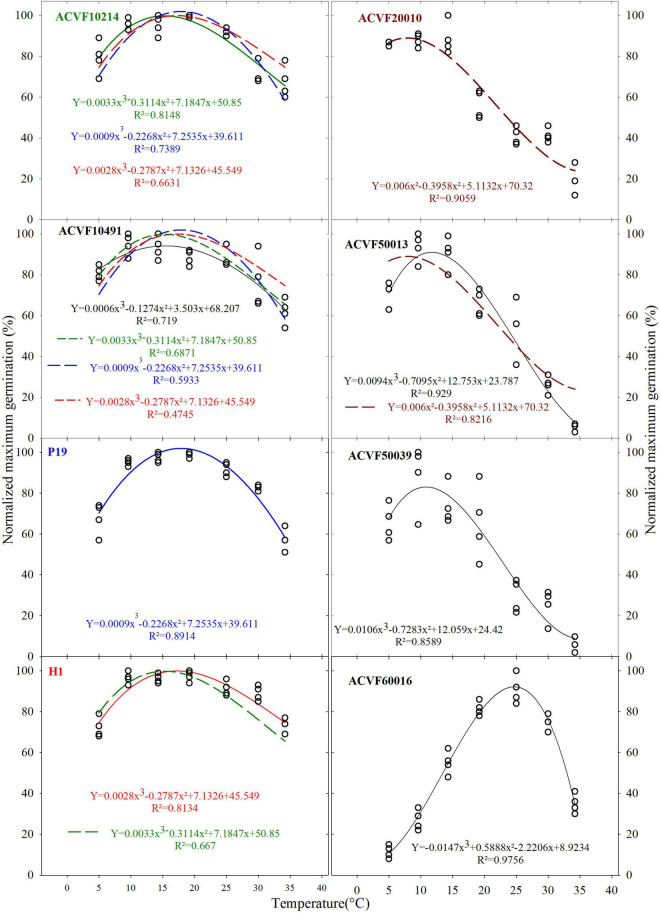
Normalized germination percentage in response to a constant temperature of eight accessions of *Lolium perenne*.

Temperature and time course response-surface plots, given in Figure 1, were constructed with the average of four replicates from the time course of germination at each temperature, for each accession. No germination was observed at 40°C for any accession, neither in the main experiment nor in the subsidiary one. Thus data equal to zero were not plotted nor used in the curve fitting. Although recordings of germination were performed for as long as 2,352 h (98 days) at 5°C, the time axis is limited to 1,920 h. These response-surface plots illustrate clearly that time course responses to temperature of the eight accessions were highly contrasted, not only in the final normalized cumulative germination percentage but also in the shape of the curves (Figure 2). On one hand side, the four accessions on top of Figure 1 (ACVF10214, ACVF10491, P19, and H1) could be considered as following a paragon with fast germination and a somehow flat response to temperatures between 10 and 25°C. On the other hand, the four accessions at the bottom (ACVF20010, ACVF50013, ACVF50039, and ACVF60016) had different germination speeds and a narrow range of temperatures to express their highest germinability.

### Comparisons of the Maximum Germination Percentage Curves

At first sight, four types of responses to temperature for germinability can be observed (Figure 2). The first type, on the left of Figure 2, maximum germination is obtained between 10 and 25°C. Small effects of extreme temperatures (5 and 35°C) are observed. The second type is represented by only one accession (ACVF20010). Unexpectedly, maximum germinability was observed at 5, 10, and 15°C. In the third type, accessions ACVF50013 and ACVF50039 were little affected by low temperature (5°C), while the temperatures for highest germinability were 10 and 15°C. For these two latter types, germinability decreases with temperature increase beyond 15°C. Finally, the response of accession ACVF60016 is very different from the previous three types. It is a very atypical right-side skewed bell, showing a clear optimum for germination at 24°C and high sensitivity to the extreme temperatures tested.

The plots in Figure 2 make explicit the variability observed on germination percentage at the eight tested temperatures for the eight accessions of *L. perenne*. For each accession, the best fitting with a third-degree polynomial is presented. Because germination was not observed for any accessions at 40°C, these data were excluded from the curve fitting. These curves were used to compare the responses of accessions. The pair-wise comparisons of normalized maximum germination percentage curves were performed based on the sequential analysis of: (i) residuals’ normality, (ii) residuals’ non-bias, and (iii) lack-of-fit probabilities.

It was observed that the responses curves of the accessions showed significant differences (*P* < 0.05). Indeed, the lack of fit tests showed that most curves are not exchangeable and thus they should be considered as being different. In fact, only five curves, out of 56 comparisons, can fit data of other accessions. This means that limited similarities between responses can be observed. Indeed, we noted, the time course of germination for the two wild populations ACVF10214 and ACVF10491, and the two selection populations P19 and H1 appear similar in response to a constant temperature between 5 and 35°C, compared with the other populations. Curves of P19 and H1 fitted significantly well the data of ACVF10214 and ACVF10491. The only reciprocity of fitting was that of ACVF10214 with H1 (Figure 2).

### Germination Rates

**FIGURE 3:**
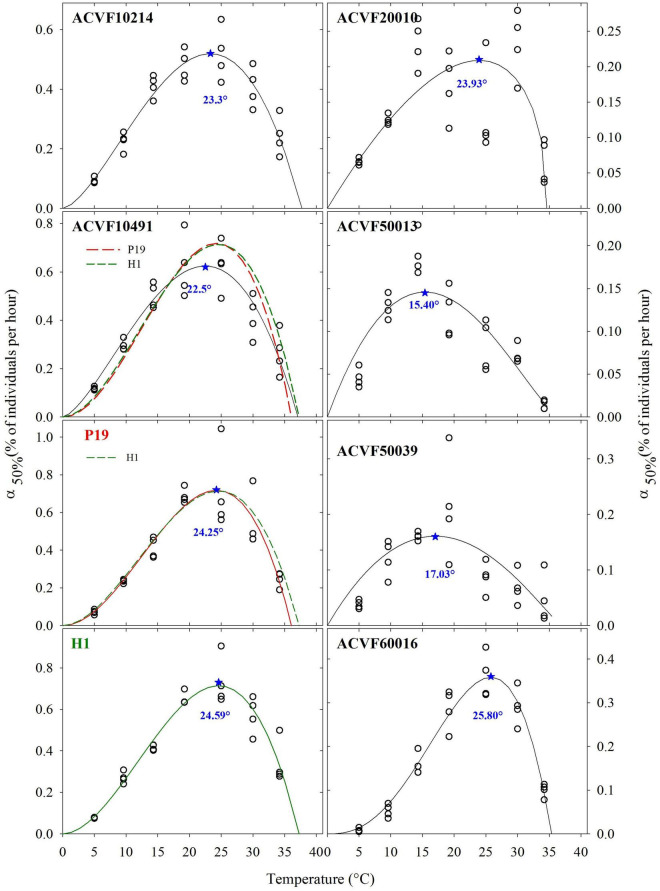
Estimated germination rates, when 50% of the seeds have germinated (α_50%_), fitted with the beta model, for eight accessions of *Lolium perenne* in response to a constant temperature *indicates the estimated optimal temperature.

The germination rates (% of seeds per hour) at the time when half of the seeds have germinated α_50%_, were fitted to a beta model (Figure 3). Furthermore, the goodness of fit was not different between accessions of the left-hand side. Thus, the sequential analysis of pair-wise comparisons detected no differences between accessions of the right side that were significant (*P* < 0.05) (Figure 3). This could be explained by the smaller variability in germination rates, α_50%_, estimates. In general, α_50%,_ increased as temperature increased from low to optimal temperature and decrease afterward. Nevertheless, high variability, and to some point, erratic variation of the estimated values were observed. Indeed, it can be said that, in general, germinations rates of the eight *L. perenne* accessions respond differently to temperature in the range 5–35°C. For curve fitting purposes, the lower boundary was fixed to 0°C. The upper limit appeared between 35 and 40°C.

**FIGURE 4:**
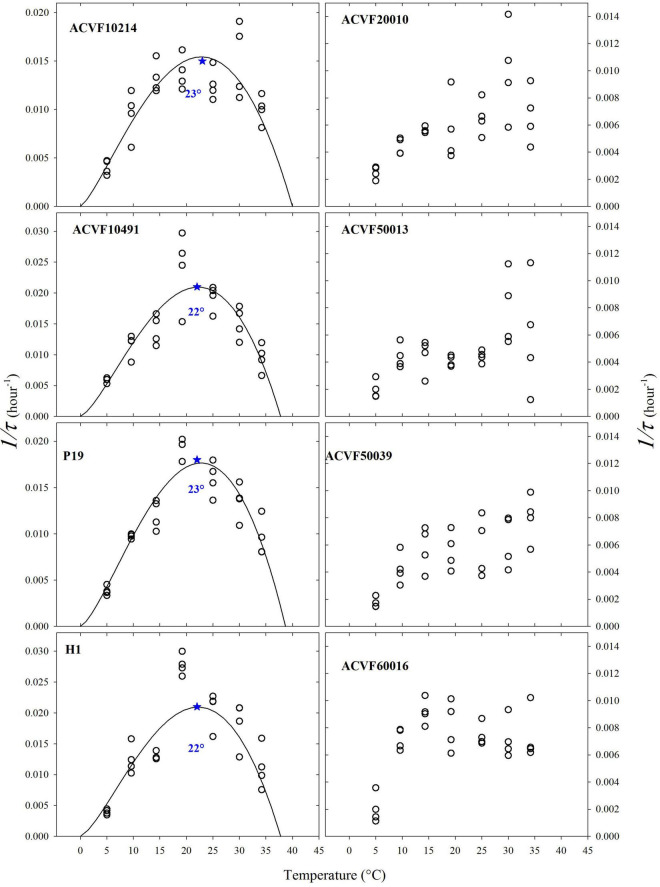
The inverse of the time (1/τ) needed to obtain 50% of the germinating seeds germinated for eight accessions of *Lolium perenne* *indicates the estimated optimal temperature.

Some authors use the inverse of the time needed to reach a given germination percentage, inverse of time for short, as an estimator of germination rate [see Ranal and Santana (2006) and references therein]. Thus, we calculated the inverse of the time (1/τ) needed to reach the point where 50% of the germinating seeds germinate (Figure 4). The resulting plots make some sense only for half of the *L. perenne* accessions. Indeed, for accessions ACVF10214, ACVF10491, P19, and H1 germination rate increased as temperature increased from the low to optimal temperature and then decreased above optimal temperature. For the other half, it was not possible to fit properly a beta function because 1/τ showed high variability at high temperatures and the germination rate did not decrease. This 1/τ parameter did not allow estimating optimal temperature to get a maximum rate of germination.

### Initial Heterotrophic Growth

We used sequential photographs to follow up, measure, and analyze the variables related to the initial heterotrophic growth summarized by root and shoot elongation.

#### Final Length of the Radicle

**FIGURE 5:**
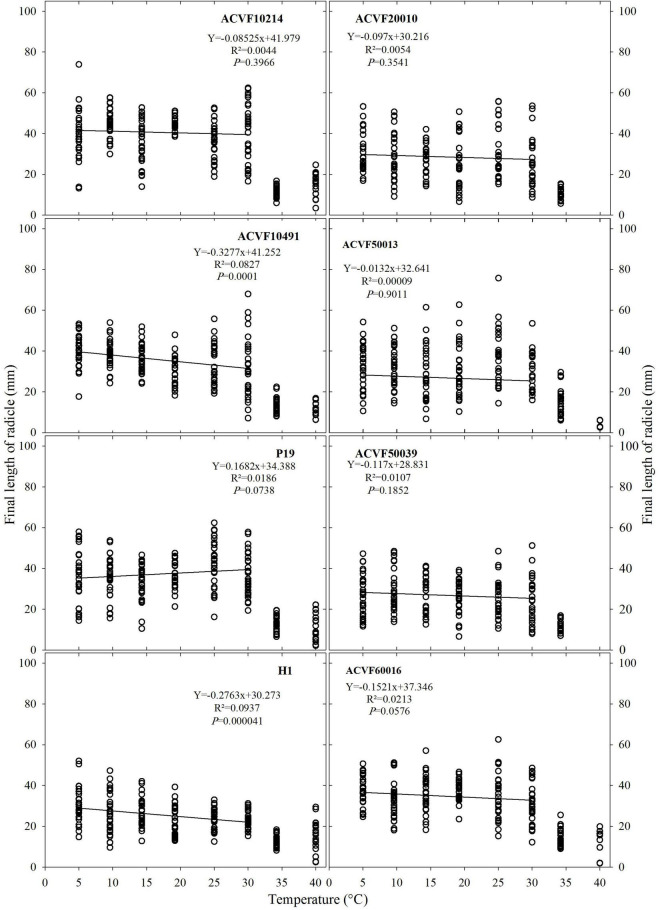
Final length of radicle for the accessions of *Lolium perenne* during heterotrophic growth in response to a constant temperature. For each temperature between 5 and 35°C, *n* = 30. For 40°C, 0 ≤ *n* ≤ 30 because of seedling mortality.

The final length of the radicle (FL-rt) of the eight accessions grown at a constant temperature ranging from 5 to 40°C is shown in Figure 5. High variability was observed within each accession and temperature. It appears clearly that at 35 and 40°C, FL-rt was smaller than the values recorded at the other temperatures. Thus, the effects of temperature on FL-rt were analyzed by linear regression for the points between 5 and 30°C. For the two wild accessions ACVF20010 and ACVF50039, the 40°C treatment was lethal. Those lots were very sensitive to extreme temperatures and theirs optimal temperatures for germination were 8 to 10°C, respectively. Only two accessions (ACVF10491 and H1) out of eight accessions responded significantly (*P* < 0.05) to temperature (slope ≠ 0). For them, FL-rt decreased as temperature increased from 5 to 30°C.

#### Final Length of the Shoot

**FIGURE 6:**
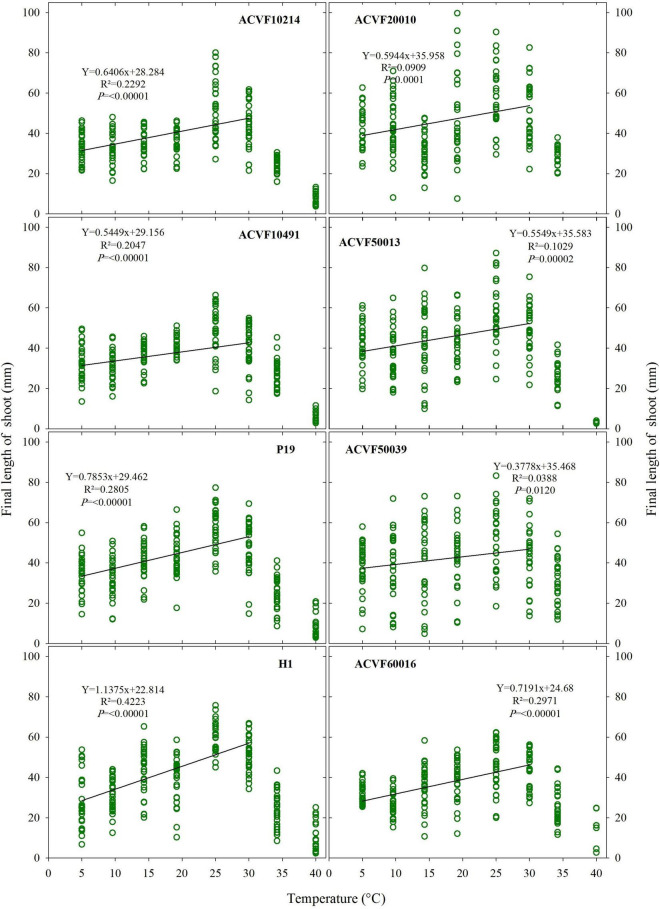
The final length of shoot for the eight accessions of *Lolium perenne* during heterotrophic growth in response to a constant temperature. For each temperature between 5 and 35°C, *n* = 30. For 40°C, 0 ≤ *n* ≤ 30 because of seedling mortality.

Like FL-rt, the final length of the shoot (FL-sh) showed high variability within each accession and temperature (Figure 6). If the data from 35 and 40°C are withdrawn, significant (*P* < 0.05) linear responses to temperature are observed (slope ≠ 0).

It appears from data in Figures 5, 6 that FL-sh was more sensitive than FL-rt to the increase of growing temperature from 5 to 30°C.

### Parameters of Schnute Equation Describing the Relative Growth Rate of Axes

Schnute non-linear equation (Equation 6) was fitted to data on the time course of growth. Its first and second derivatives were used to estimate absolute growth rates while Equation 7 allowed estimating the relative growth rate of the radicle (RGR-rt) and shoot (RGR-sh).

Curve fitting to data from 40°C yielded poor results because this temperature appeared lethal to most seedlings. Furthermore, under other temperatures, in some cases, followed seedlings were abnormal, showing no growth of the radicle or the shoot. Thus, estimated parameters in both situations are not presented hereafter because they were not analyzed.

#### Relative Growth Rate of Radicle

**FIGURE 7:**
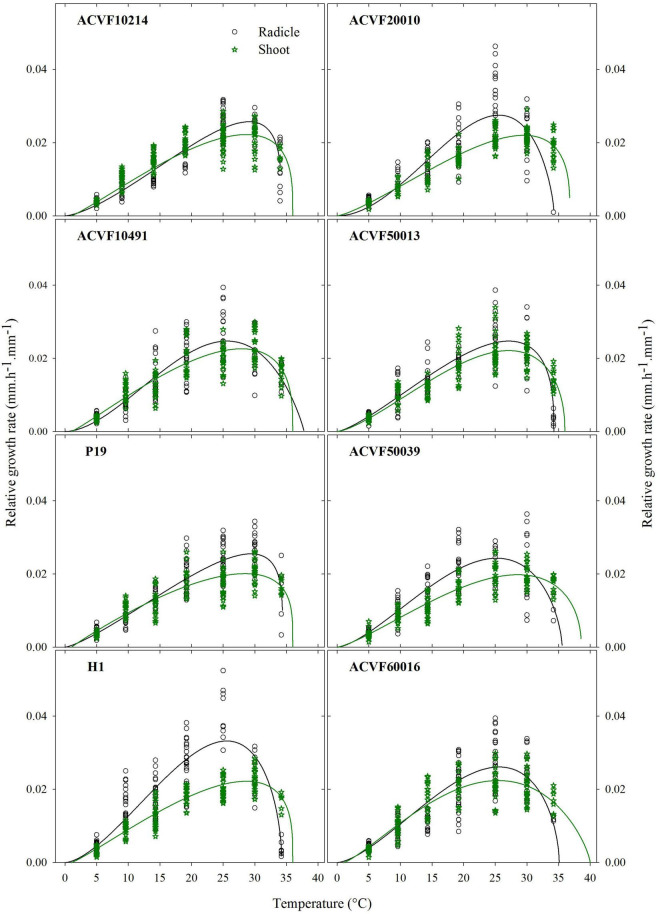
Relative growth rate of radicle and shoot were observed for eight accessions of *Lolium perenne* during heterotrophic growth in response to a constant temperature. For each temperature between 5 and 35°C, *n* = 30. For 40°C, 0 ≤ *n* ≤ 30 because of seedling mortality.

For each accession, the best fitting with a beta equation (Equation 5) is presented in Figure 7. These curves were used to compare the responses of accessions to constant temperature from 5 to 35°C. Cardinal temperatures and relative growth rates were estimated for each accession (Table 3). For curve fitting purposes, the lower and upper boundaries were fixed at 0 and 40°C, respectively.

**TABLE 3:** Estimated values (*Pr* > *t*) of the parameters for the beta model fitted to the relative growth rate of the radicle RGR-rt data of *Lolium perenne* accessions in response to constant temperature during heterotrophic growth.

Accession	RGR (mm.h^–1^.mm^–1^)	*T*_*min*_ (°C)	*T*_*o*_ (°C)	*T*_*max*_ (°C)	δ
ACVF10214	0.0257 ± 0.0009	0 ± 3.5	29.1 ± 1.3	34.2 ± 0.4	0.2601 ± 0.15
ACVF10491	0.0247 ± 0.0009	0 ± 5.4	25.6 ± 1.3	37.8 ± 5.1	0.8746 ± 0.86
P19	0.0255 ± 0.0007	0 ± 4.0	29.4 ± 1.4	34.4 ± 0.4	0.2211 ± 0.15
H1	0.0332 ± 0.0014	0 ± 3.9	25.6 ± 0.8	34.3 ± 0.1	0.5539 ± 0.20
ACVF20010	0.0275 ± 0.001	0 ± 4.2	25.6 ± 0.6	34.3 ± 0.2	0.6749 ± 0.20
ACVF50013	0.0247 ± 0.0009	0 ± 4.0	27.2 ± 1.3	34.3 ± 0.1	0.3559 ± 0.17
ACVF50039	0.0243 ± 0.001	0 ± 4.7	25.2 ± 1.1	35.6 ± 1.9	0.6237 ± 0.39
ACVF60016	0.0261 ± 0.0008	0 ± 4.5	26.6 ± 0.8	35.1 ± 1.1	0.5792 ± 0.28

*RGR is the relative growth rate of the accession; T_min_, T_o_, and T_max_ are the cardinal temperatures; and δ is a unitless shape parameter.*

In general, RGR-rt was slow at 5°C, peaked between 25 and 29°C, and showed a sharp reduction afterward. Accession H1 had the fastest radicle growth rate (0.033 mm.h^–1^. mm^–1^) whereas ACVF50039 had the slower one (0.024 mm.h^–1^. mm^–1^).

Relative growth rate of radicle (RGR-rt) curves were used to perform comparisons between accessions based on a test of lack of fit between the best beta model of one accession and the data from another one. This lack of fit test includes a normality test and a bias test of the residuals (data not shown). Significant differences (*P* < 0.01) in RGR-rt were observed between accessions. Indeed, the RGR-rt of selection accession (H1) is very different from the other accessions. Regardless of the accession H1, data show high variability between 10 and 30°C. However, some accessions have exchangeable curves. Noteworthy, the range of optimum temperature is 25.2–29.4°C. The upper boundary, *T*_*max*_, was estimated between 34.2 and 37.8°C which is lower than for the shoot (see below).

#### Relative Growth Rate of Shoot

The relative growth rates of the shoot (RGR-sh) of each accession were also fitted to a beta model (Figure 7). The range of optimal temperatures was 25.5–29.5°C (Table 4). The *T*_*max*_ estimated from the model was between 36 and 40°C with 36 being the most common value. Accession of ACVF50039 had the lowest RGR-sh (0.019 mm.h^–1^.mm^–1^).

**TABLE 4:** Estimated values of the parameters for the beta model fitted to the relative growth rate of the shoot (RGR-sh) data of *Lolium perenne* accessions in response to constant temperature during heterotrophic growth.

Accession	RGR (mm.h^–1^.mm^–1^)	*T*_*min*_ (°C)	*T*_*o*_ (°C)	*T*_*max*_ (°C)	δ
ACVF10214	0.0222 ± 0.0004	1 ± 2.5	28.6 ± 1.0	36.0 ± 2.0	0.3039 ± 0.19
ACVF10491	0.0226 ± 0.0005	1 ± 3.2	27.9 ± 1.2	36.0 ± 2.3	0.3385 ± 0.25
P19	0.0201 ± 0.0005	1 ± 3.5	28.5 ± 1.5	36.0 ± 2.9	0.2663 ± 0.25
H1	0.0222 ± 0.0004	1 ± 2.5	28.6 ± 1.0	36.0 ± 2.0	0.3039 ± 0.19
ACVF20010	0.0220 ± 0.0004	0 ± 3.5	29.5 ± 1.0	36.8 ± 3.1	0.3223 ± 0.27
ACVF50013	0.0221 ± 0.0005	0 ± 3.7	27.1 ± 0.9	36.0 ± 1.7	0.4656 ± 0.26
ACVF50039	0.0198 ± 0.0004	0 ± 3.8	28.6 ± 1.1	38.6 ± 5.7	0.4577 ± 0.48
ACVF60016	0.0224 ± 0.0005	1 ± 3.5	25.5 ± 1.1	40.0 ± 7.0	0.7603 ± 0.72

*T_min_, T_o_, and T_max_ are the cardinal temperatures; and δ is a unitless shape parameter.*

The response curves of RGR-sh from some accessions have exchangeable curves. Indeed, the lack-of-fit test showed that the curve of population selection (P19) could fit of accession ACVF50039. Furthermore, the models of accessions ACVF20010 and ACVF50013 adjusted on the data of accession ACVF10214. Finally, the response of accessions ACVF10491 and ACVF60016 adjusted on the data of accessions ACVF10214 and H1.

#### Relative Growth Rate of Radicle vs. Relative Growth Rate of Shoot

Significant differences (*P* < *0.01*) were observed between the relative growth rates of the radicle and the shoot only for wild accessions ACVF20010 and ACVF50039 and for breeding populations P19 and H1 (Figure 7).

## Discussion

Studying the effects of temperature on plant physiology is a topical issue, which has received considerable attention in recent years. A great part of the studies reported in the scientific literature concerns seed germination. Germination is a complex biological process influenced by genetic and environmental factors including temperature (Shafii and Price, 2001). However, the genetic diversity of response to temperature during seed germination of pasture species has received little attention (Volaire et al., 2016; Ahmed et al., 2019; Ghaleb et al., 2021, 2022). A similar situation is observed for the study of initial heterotrophic growth in general (Escobar-Gutiérrez et al., 1998a,b) and of pastures in particular (Ahmed, 2015).

### Genetic Diversity of Perennial Ryegrass in Response to Temperature During Germination

This study on *L. perenne* indicates that genetic diversity exists in germination responses to constant temperatures between 5 and 40°C. Furthermore, accessions exhibited contrasted patterns of response. Seed germination has been studied under both the constant and alternating temperatures. In many species, alternating temperatures favor germination (Baskin and Baskin, 2014). Concerning *L. perenne*, studies have been conducted to analyze the responses to constant or alternative temperatures between commercial varieties (Larsen and Bibby, 2005; Shen et al., 2008; Zhang et al., 2013). Nevertheless, reports are not conclusive on this point. For example, Danneberger et al. (1992) showed that *L. perenne* germinated well at alternating temperatures 15/35°C. Similar results are reported for two cultivars by Shen et al. (2008). However, Thompson and Grime (1983) found that germination of perennial ryegrass’ variety S22 was insensitive to the presence or absence of both light and temperature fluctuations. Furthermore, Shen et al. (2008) reported poor germination for two cultivars under a high number of combinations of two temperatures alternating from 5 to 40°C in 16/8 or 8/16 h cycles.

In this work, no germination was observed for any populations at 40°C. Already at 35°C, germination was affected. Monks et al. (2009) reported that the *L. perenne* variety Commando fully germinated at constant temperatures between 5 and 30°C, but did not germinate at 35°C. Likewise, in the work of Zhang et al. (2013), germination was not observed at 37.5 and 40°C whereas germination was only 1% at 35°C for a variety of *L. perenne*. Under constant temperature treatment, Shen et al. (2008) reported a decrease in germination percentage from 30°C and observed 15 and 30% germination at 40°C.

Low temperature can also affect the germination percentage of varieties or accessions. In this work, in some cases (ACVF20010, ACVF50013, and ACVF50039) low temperature favors cumulated germination percentage, but in other cases, the opposite was observed. For example, the most favorable temperature for wild accessions ACVF20010, collected in the Mediterranean area, was 7.9°C. Our experiments at 5°C were conducted for a period long enough to observe germination reaching plateaus for every accession. On the other hand, one can wonder if the low germination percentages at low temperature reported by Larsen and Bibby (2005); Shen et al. (2008), and Zhang et al. (2013) resulted from too early final samplings. Yoon et al. (1985) reported that the optimal temperature for germination rate was 15 and 25°C, while Lodge (2004) reported a maximum germination rate of this species at 5/15–30/35°C alternating diurnal temperature cycles for *L. perenne*. Other species of the genus *Lolium* have optimal temperatures in similar ranges as reported by Turner et al. (2001) for *L. rigidum* and by Hill et al. (1985) for *L. multiflorum*.

Geographically related variation in germination response is common among widespread species (Lord, 1994). However, the genetic diversity of *L. perenne* in response to alternating or constant temperature during germination has not been explored concerning the provenance of accessions. Our hypothesis concerning the genetic diversity in response to constant temperature during germination was confirmed.

The temperature had a marked effect on the timing of germination (parameters *tc*, *t*_50%_, and **τ** in Equation 1, data not shown) and the germination rates (% of seeds per hour). We observed differences for all these parameters between the eight *L. perenne* accessions. It took a long time to get the maximum cumulated germination percentage at extremes temperatures of 5 and 35°C. From an agronomical perspective, accessions or varieties presenting low timing lags between imbibition of the dry seed and emergence and high germination rate could be interesting for early sowing to favor seedling establishment well before the summer droughts start (Black et al., 2006; Monks et al., 2009; Zhang et al., 2013).

### Genetic Diversity of Perennial Ryegrass in Response to Temperature During the Initial Heterotrophic Growth

The final length of roots and shoots were both sensitive to temperature with opposite responses between 5 and 30°C. In this regard, Lawlor (1987) showed that cardinal temperatures for germination were different from those for growth of both seedling axes: radicle and coleoptile.

Numerous studies have explored the relationships between temperature and seedling growth in *Zea mays* (Grobbelaar, 1963; Blacklow, 1972), *Gossypium hirsutum* L. (Arndt, 1945), and *Pennisetum americanum* L. (Carberry and Campbell, 1989), but they have not explored a wide range of genetic variation. The wild accessions and varieties that we studied have very different genetic origins. Our accessions are characterized by different maximum RGR of the root and the shoot in response to temperature.

The response of relative growth rate of either radicle or shoot to temperature did differ between some varieties (e.g., H1). Furthermore, the maximum RGR-rt for some accessions was significantly different from the maximum RGR-sh. The most favorable temperatures to reach the maximum RGR of the radicle and shoot were between 25 and 30°C, which are compatible with published data (Beard, 1973; Simon, 1981; Masiunas and Carpenter, 1984).

Similar to germination, our hypothesis concerning the genetic diversity in response to constant temperature during initial heterotrophic growth was confirmed.

## Conclusion

Studying the effects of temperature on plant physiology is a topical issue. Nevertheless, literature on *L. perenne* is relatively scarce. Our accessions showed significant differences in their responses. Even in a saturated atmosphere, 40°C appears as an upper thermal limit for germination and initial heterotrophic growth of *L. perenne*.

The novelty of this study comes from the wide range of temperatures evaluated. Furthermore, this is the first work showing that seed germinability of wild populations from northern and cold sites is enhanced by high temperatures and limited by colder temperatures and vice versa for warm-adapted populations from the South.

We analyzed a small sample of *L. perenne* germplasm for the response to constant temperature during germination and initial heterotrophic growth. Considering the vast natural genetic diversity of resources of *L. perenne* recently unearthed (Blanco-Pastor et al., 2019; Keep et al., 2020), increasing the sample size of accessions should confirm the results presented here and certainly reveals unseen responses to constant temperatures during germination and initial growth. These findings and those presented in this article prompted us to to initiate an ongoing work where 373 seed lots of *L. perenne* are under study.

The results presented here reveal, for the first time, high genetic variability within *L. perenne* germplasm for the response to temperature in the initial life phases. We have recently shown that the response of germination to temperature is at least partially controlled genetically (Ghaleb et al., 2022). Thus, the newly discovered genetic variability should serve breeders to create perennial ryegrass varieties for the future.

## Data Availability Statement

The raw data supporting the conclusions of this article will be made available by the authors, without undue reservation.

## Author Contributions

AE-G designed the research and secured funding. LA and AE-G conducted the experiment, analyzed the data, and wrote the manuscript. Both authors approved the final version of the manuscript.

## Conflict of Interest

The authors declare that the research was conducted in the absence of any commercial or financial relationships that could be construed as a potential conflict of interest.

## Publisher’s Note

All claims expressed in this article are solely those of the authors and do not necessarily represent those of their affiliated organizations, or those of the publisher, the editors and the reviewers. Any product that may be evaluated in this article, or claim that may be made by its manufacturer, is not guaranteed or endorsed by the publisher.

## Footnotes

1
http://florilege.arcad-project.org/fr/crb/especes-fourrageres

2
http://imagej.nih.gov/ij/

3
https://www.r-project.org
